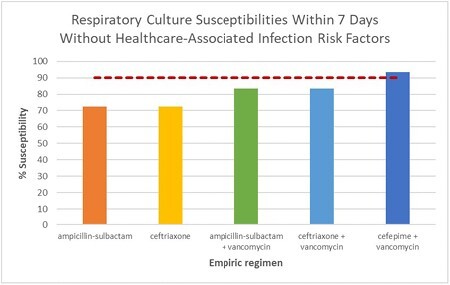# 118 Retrospective Analysis of Pathogens for Guided Creation of an EMPIRic Antibiotic PrEscribing Pathway (EMPIRE)

**DOI:** 10.1093/jbcr/irae036.117

**Published:** 2024-04-17

**Authors:** Lorraine A Todor, David M Hill

**Affiliations:** Regional One Health, Memphis, TN; Regional One Health, Memphis, TN

## Abstract

**Introduction:**

Mortality from infection due to P. aeruginosa is remarkably high; therefore empiric regimens include its coverage. In a prior study, we showed manual inclusion of bedside prescribing considerations heavily reduced antibiogram-based, empiric recommendations (i.e, double Gram-negative [GN] coverage to a single agent + vancomycin [VAN]). The purpose of this study is to determine if empiric regimens in the burn unit may be optimized further per suspected infection source. We hypothesized an initial regimen of VAN and cefepime (C4) will still adequately cover 90% of definitive pathogens isolated within one week of admission in absence of healthcare-associated infection risk factors (HAIRF). For suspected pneumonia, a narrower beta-lactam can be used with adequate coverage. Lastly, difficult to treat pathogens (DTp) are rarely the cause of wound infection within one week of admission.

**Methods:**

This single-center, retrospective study included patients admitted to the burn unit between January 1, 2020 and December 31, 2022. Patients were screened by reviewing microbiology reports during the study period and exclusion criteria applied to generate a final sample of patients and cultures. Only treated infections were considered (i.e., not surveillance data). A 3-year sample was utilized to ideally achieve 30 isolates of the most common pathogens. Demographic data was reported using descriptive statistics. Sensitivities were reported via antibiotic*pathogen and cumulatively according to source and hypothetical future regimen.

**Results:**

The final sample included 268 pathogens. Tissue samples from wounds, respiratory samples, and blood accounted for 183 (68%) 35 (13%), and 30 (11%) culture sources, respectively. Urine and bone samples made up less than 8% of pathogens. VAN and C4 covered 98% and 90% of Gram-positive and GN pathogens, respectively, within the first week of admission and in absence of HAIRF. Fifteen respiratory cultures grew GN pathogens, of which 83% were susceptible to C4. When used in combination with VAN, 93% of all respiratory pathogens that grew were covered. Narrower beta-lactams, ampicillin-sulbactam or ceftriaxone, plus VAN covered 83% of respiratory pathogens. Of the 183 wound cultures collected, 10 (5.5%) DTp were isolated.

**Conclusions:**

Despite recommendations for empiric double coverage of GNs per the local unit-specific antibiogram, initial use of VAN and C4 remains adequate for pathogens isolated within one week of admission in patients without HAIRF. For pneumonias, a narrower spectrum beta-lactam would not sufficiently cover respiratory pathogens isolated within the first week of admission. DTp are rarely isolated from wound cultures within one week of admission.

**Applicability of Research to Practice:**

Antibiotics must be used judiciously to combat antibiotic resistance. We describe a method to consider for creating empiric prescribing pathways for adequate pathogen coverage while preserving broader agents.